# Carbon Paste Electrodes Made from Different Carbonaceous Materials: Application in the Study of Antioxidants

**DOI:** 10.3390/s110201328

**Published:** 2011-01-25

**Authors:** Constantin Apetrei, Irina Mirela Apetrei, Jose Antonio De Saja, Maria Luz Rodriguez-Mendez

**Affiliations:** 1 Department of Chemistry, Faculty of Sciences, Dunărea de Jos University of Galaţi, 800008 Galaţi, Romania; E-Mail: apetreic@ugal.ro; 2 Condensed Matter Physics Department, Faculty of Science, University of Valladolid, 47011 Valladolid, Spain; E-Mails: irina_apetrei@yahoo.com (I.M.A.); sajasaez@fmc.uva.es (J.A.S.); 3 Department of Inorganic Chemistry, Escuela de Ingenierías Industriales, University of Valladolid, 47011 Valladolid, Spain

**Keywords:** carbon paste electrode, carbonaceous material, carbon nanotube, cyclic voltammetry, antioxidant

## Abstract

This work describes the sensing properties of carbon paste electrodes (CPEs) prepared from three different types of carbonaceous materials: graphite, carbon microspheres and carbon nanotubes. The electrochemical responses towards antioxidants including vanillic acid, catechol, gallic acid, l-ascorbic acid and l-glutathione have been analyzed and compared. It has been demonstrated that the electrodes based on carbon microspheres show the best performances in terms of kinetics and stability, whereas G-CPEs presented the smallest detection limit for all the antioxidants analyzed. An array of electrodes has been constructed using the three types of electrodes. As demonstrated by means of Principal Component Analysis, the system is able to discriminate among antioxidants as a function of their chemical structure and reactivity.

## 1. Introduction

Oxidative stress produces damage to lipids, proteins, DNA and small cellular molecules impeding normal cell functioning. These biochemical alterations are implicated in a growing list of human diseases, such as cardiovascular diseases, aging, Parkinson’s disease, Alzheimer’s disease, diabetes and cancer [1]. Antioxidants are compounds that inhibit or delay the oxidation process by blocking the initiation or propagation of oxidizing chain reactions. They may function as free radical scavengers, complexers of pro-oxidant metals, reducing agents and quenchers of singlet oxygen [2–4].

Several antioxidant agents that include vitamins, glutathione as well as a range of polyphenols are found in wines [5–8]. Phenolic compounds are a complex group of substances that have attracted considerable attention due to their role in providing a characteristic flavor and color to wines and because their benefits to human health which are associated with their antioxidant activities [9,10]. Other antioxidants of interest present in wines are the ascorbic acid which is one of the most ubiquitous vitamins ever discovered and simultaneously, one of the most beneficial antioxidants [11,12] and glutathione, a major intracellular thiol compound that plays an important role in many biological processes such as intracellular reduction-oxidation metabolic cycles, transportation, protein synthesis, catabolism, and metabolism [2,4].

Among the wide variety of techniques used to detect antioxidants, electrochemical methods have the advantage of their high sensitivity, low cost and inherent portability [13]. Carbon paste electrodes (CPEs) are widely applicable in electrochemical studies due to their low background current (compared to solid graphite or noble metal electrodes), low cost, feasibility to incorporate different substances during the paste preparation (in the case of modified carbon paste electrodes), easy preparation, simple renewal of their surface and possibilities of miniaturization. Different types of carbonaceous materials (graphite, glassy carbon, acetylene black, diamond, carbon nanofibers, carbon microspheres and carbon nanotubes) have been employed to construct CPEs using a variety of methods [14–18]. It is well known that compounds which are antioxidants by virtue of their ability to act as reductants, can be easily oxidized at unmodified and chemical modified electrode surfaces. CPEs have been used as the working electrode in cyclic voltammetry experiments aimed to the identification, characterization and quantification of antioxidants, including ascorbic acid [19], phenolic and polyphenolic compounds [20–23], glutathione [24] and synthetic antioxidants [25].

In spite of the number of works were CPEs have been used to detect antioxidants, the influence of the carbonaceous material in the performance of the electrodes has not been sufficiently explored. Moreover, the sensing characteristics of CPEs prepared using different carbonaceous materials have not been compared. In this paper, carbon paste electrodes based in three carbonaceous materials (graphite, carbon microspheres and carbon nanotubes) have been prepared and their capability to detect antioxidants present in wines has been compared. For this purpose, the surface areas of the electrodes have been determined by cyclic voltammetry using a potassium ferrocyanide solution. Then, cyclic voltammetry has been applied to evaluate the responses of the electrodes towards five antioxidants including ascorbic acid, glutathione, vanillic acid (a monophenol), catechol (a diphenol) and gallic acid (a triphenol). The electrochemical behavior of the electrodes has been analyzed and the kinetics and the sensitivities have been discussed. The discrimination capacity of the electrodes among antioxidants has been demonstrated by means of Principal Component Analysis (PCA).

## 2. Experimental Section

### 2.1. Apparatus

All the voltammetric measurements were carried out in a 100 mL thermostated glass cell at 25 °C, in a three-electrode configuration. Carbon paste electrode was used as a working electrode. The reference electrode was an Ag|AgCl/KCl_sat_ and the counter electrode was a platinum plate. Cyclic voltammetric measurements were performed in an EG&G PARC Model 263 potentiostat/galvanostat (Princeton Applied Research Corp., NJ, USA) connected to a desktop computer and controlled by a software (Echem). Cyclic voltammograms were registered from −0.5 to +1.3 V at a sweep rate of 0.1 V·s^−1^ (except otherwise indicated).

### 2.2. Reagents and Solutions

**Figure 1.**
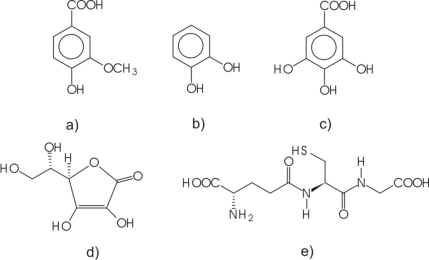
Chemical structure for vanillic acid **(a)**; catechol **(b)**; gallic acid **(c)**; l-ascorbic acid **(d)**; l-glutathione **(e).**

All the solutions were prepared using water purified in Millipore Milli-Q system. All chemicals were of analytical grade and used without further purification. Gallic acid and reduced l-glutathione were purchased from Sigma-Aldrich. Other purchased chemicals included l-ascorbic acid (Riedel-de Haën), vanillic acid (Fluka), and catechol (Panreac). The chemical structures for these antioxidants are given in Figure 1.

A model solution of wine was prepared consisting of 12% (v/v) ethanol (Panreac), 0.05 mol·L^−1^ l-(+)-tartaric acid (Sigma), and added NaOH (Panreac) to give a pH of 3.6. Additionally, an aqueous solution 0.1 mol·L^−1^ of KCl was used as a reference solution. The solutions of antioxidants were prepared by dissolving the corresponding antioxidant in the model solution of wine in a concentration of 4 × 10^−4^ mol·L^−1^ except otherwise indicated.

### 2.3. Carbon Paste Electrode Construction

Graphite powder (High purity Ultracarbon^®^, Ultra F purity. Bay City, MI, USA), carbon microspheres (from Sigradur G HTW, Thierhaupten, Germany), carbon nanotubes (multi wall nanotubes, Nanoledge Inc., Boncherville, Quebec, Canada) and high purity mineral oil (Nujol, Fluka) were used in the preparation of the carbon paste.

**Figure 2.**
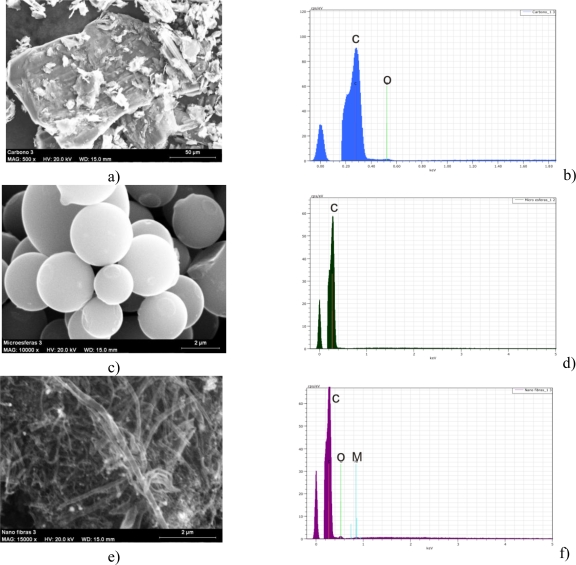
SEM images of the carbonaceous materials used for the electrode construction: (**a**) graphite; (**b**) carbon microspheres; (**c**) multiwall carbon nanotubes. EDAX analysis for: (**d**) graphite; (**e**) carbon microspheres and (**f**) multiwall carbon nanotubes.

The carbon paste electrodes were prepared by mixing the carbonaceous material with a binder (Nujol) and the blend was mixed until a homogenous paste with the appropriate consistence was obtained. In order to prepare a paste able to be compactable different ratios carbon:Nujol were used for each type of carbonaceous material: 1:1.5 (w:w) for graphite (G), 1:1.6 (w:w) for carbon microspheres (μS) and 1:2 (w:w) multiwall carbon nanotubes (CNT). The carbonaceous materials were characterized by SEM (JEOL JSM-820 scanning microscope) and EDAX (Bruker AXS XFlash Detector 4010). The SEM images and the results of the EDAX analysis are presented in Figure 2.

The EDAX analysis confirmed the high purity of the carbonaceous materials used for electrode preparation. Once prepared, 0.1 g of the mixture were introduced in a plastic syringe (1 mL), and compressed. Appropriate packing was achieved by pressing the electrode surface against a filter paper. A copper wire was used as a contact. The CPEs were finally smoothed manually by a clean filter paper.

### 2.4. Chemometrics

A non-supervised multivariate method such as principal component analysis (PCA) was used for the analysis of the electrochemical signals obtained from the antioxidant solutions. In order to obtain six replicates of each sample, six antioxidant solutions (4 × 10^−4^ mol·L^−1^) were prepared for each antioxidant. Then, the CVs were registered in a random order using all CPEs. The voltammetric signals were preprocessed using kernel method [14,21], that was carried out using the software Matlab v5.3 (The Mathworks Inc., Natick, MA, USA). The voltammograms were pre-processed using the adaptation of a data reduction technique based on predefined response “bell shaped-windowing” curves called “kernels” [14,21]. The CV curve (i *vs*. E) is divided into anodic and cathodic branch and the anodic part is multiplied by a number of 10 smooth, bell-shaped-windowing functions, and integrated with respect to potential. Using this method, ten parameters per voltammogram were obtained and used the input variable for statistical analysis. The PCA was performed by using the software The Unscrambler 9.1 (CAMO, Oslo, Norway).

## 3. Results and Discussion

### 3.1. Electrochemical Properties of the CPEs in Electroinactive Solutions

**Figure 3.**
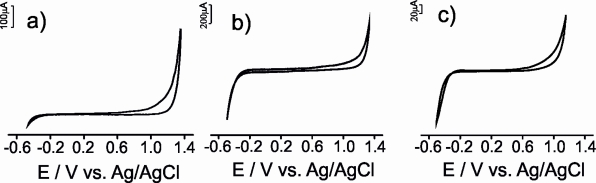
The voltammetric responses of G-CPE **(a)**; μS-CPE **(b)** and CNT-CPE **(c)** immersed in aqueous 0.1 mol·L^−1^ KCl solution.

The voltammetric behavior of the carbon paste electrodes was investigated in 0.1 mol·L^−1^ KCl solution. As expected, no electrochemical peaks were observed in the potential range studied (from −0.5 V to 1.3 V) (Figure 3).

The CVs were characterized by low background currents that decreased in the order μS-CPE > G-CPE > CNT-CPE. The current generated could be due, to capacitive effects [26]. Similar results were obtained when the electrodes were immersed in the model solution of wine that simulates the electrolytic media found in wines. This result demonstrates that the model wine solution can be successful used as supporting electrolyte for the antioxidant electroanalysis.

### 3.2. Determination of the Surface Area of the Electrodes

The surface area of the CPEs was determined by cyclic voltammetry using 1 × 10^−3^ mol·L^−1^ solution of potassium ferrocyanide in 1 mol·L^−1^ potassium chloride solution. Cyclic voltammetric responses were registered at different scan rates (0.01–1.0 Vs^−1^). Representing the anodic peak current versus the square root of the scan rate, a linear dependence was obtained. Therefore, the oxidation of ferrocyanide ion at CPE is diffusion controlled.

The surface area of the CPEs was determined by using Randles-Sevcik equation:

(1) $$i_{p}=2.687\cdot 10^{5}\cdot n^{3/2}\cdot A\cdot D^{1/2}\cdot \nu^{1/2}\cdot C$$

where i_p_ is the peak current in Amperes, A is the area of the electrode in cm^2^, C is the concentration of electroactive species in mM, D is the diffusion coefficient in cm^2^·s^−1^ and ν is the sweep rate in V·s^−1^ [27].

Taking into account that the diffusion coefficient for 1.0 × 10^–3^ mol·L^−1^ ferrocyanide is 7.26 × 10^–6^ cm^2^·s^–1^ in 1.0 mol·L^−1^ KCl [28], the surface area of the carbon paste electrodes could be calculated from the slope of the i_p_ *vs. v*^1/2^ plot. The surface areas of the carbon paste electrodes were found to be 8.66 × 10^−2^ cm^2^ for G-CPE, 1.67 × 10^−1^ cm^2^ for μS-CPE, and 7.82 × 10^−2^ cm^2^ for CNT-CPE, the highest value being obtained for μS-CPE. The results are in agreement with the higher background current observed for μS-CPE. The reproducibility of the surface areas determined experimentally was 3.4% for μS-CPE, 3.8% for G-CPE and 4.5% for CNT-CPE. As it will be commented in the next section, for all antioxidants analyzed the intensity of the peaks associated to the antioxidants decrease in order μS-CPE > G-CPE > CNT-CPE.

### 3.3. Electrochemical Response of the CPE Electrodes towards Antioxidants

The electrochemical response of a range of phenolic antioxidants commonly found in wines, along with ascorbic acid and glutathione were examined at the different carbon paste electrodes.

**Figure 4.**
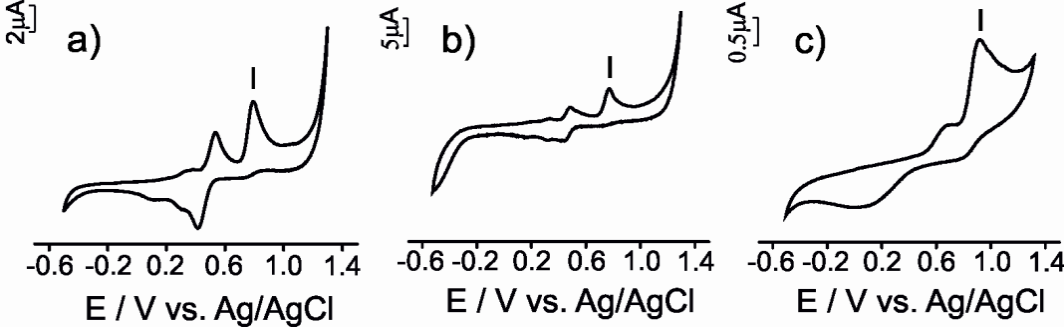

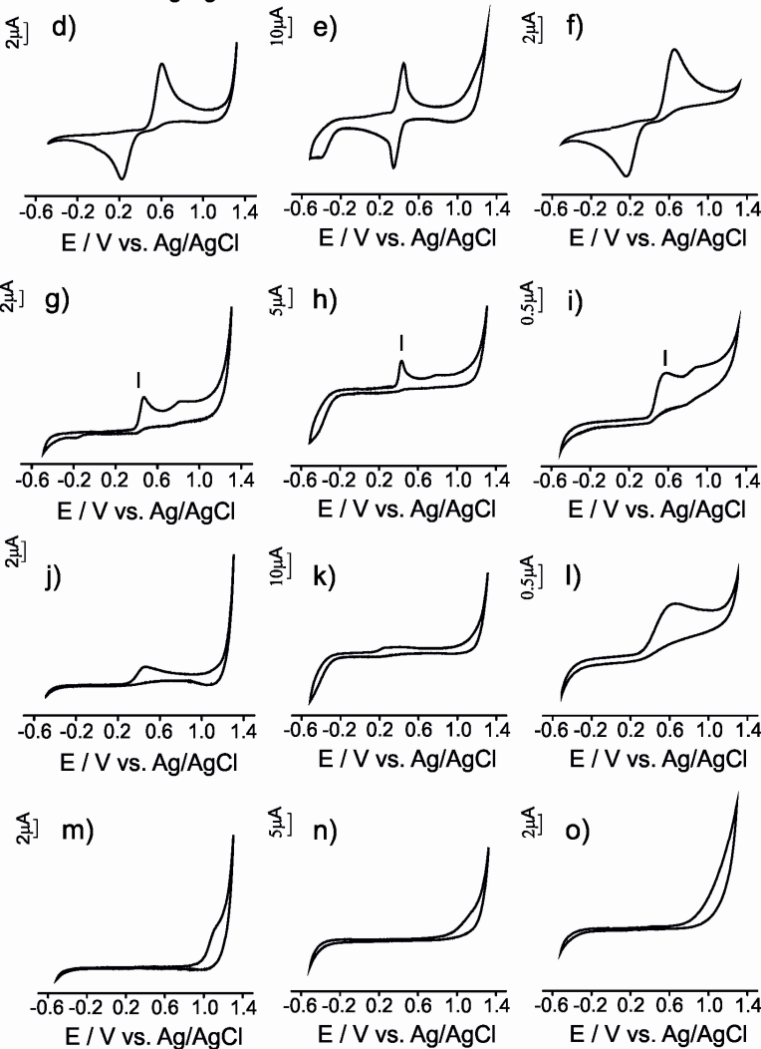
The voltammetric signals of G-CPE (left), μS-CPE (middle) and CNT-CPE (right) immersed in vanillic acid—**(a)**, **(b)** and **(c)**; catechol—**(d)**, **(e)** and **(f)**; gallic acid—**(g)**, **(h)** and **(i)**; ascorbic acid—**(j)**, **(k)** and **(l)**; glutathione—**(m)**, **(n)** and **(o)**.

The electrochemical responses obtained using the CPEs for all the antioxidant solutions studied is illustrated in Figure 4. The electrochemical parameters extracted from the responses of the three types of electrodes are summarized in Table 1.

### 3.3. Description of the Electrochemical Responses towards Antioxidants of G-CPE Electrodes

The three CPEs used in this work, produced a similar response towards vanillic acid (a mono-phenol). The first cycle was different from the subsequent ones and consisted in one anodic broad peak and some small cathodic peaks. These cathodic processes are due to the reduction of various oxidation products, some of which may remain adsorbed on the carbon electrode as an electrode film [29].

In subsequent scans two redox processes were observed (Figures 4 a,b,c). The first one at ca. E_1/2_ 0.4 V accomplished the reversibility criteria for μS-CPE and G-CPE (ΔE and |E_pa_ − E_pa/2_| were close to 0.059 V), whereas the CNT electrode showed an important degree of irreversibility indicated by the larger separation between the anodic and the cathodic wave. The second process showed an intense anodic wave accompanied by a weak cathodic process with an i_pc_/i_pa_ ratio clearly higher than one. The difference in intensity between the anodic and the cathodic wave is more marked in CNT-CPE, confirming the higher degree of irreversibility shows by the CNT-CPE electrode. The difference between the first and subsequent scans indicates that the oxidation product formed during the first cycle undergoes a further chemical reaction with the formation of new electroactive species [29].

Voltammograms obtained when the electrodes were immersed in catechol (a diphenol) gave rise to two redox peaks associated with the formation of the o-quinone in a two electron and two proton reversible process (Figures 4 d,e,f) [30]. In all cases, the i_c_/i_a_ ratio was close to 1, suggesting a quasi-reversible behavior. The E_1/2_ values calculated were similar for all CPEs (E_1/2_ ca. 0.4 V). In all cases the difference between anodic and cathodic peak (ΔE) was larger than the theoretical value calculated for a totally reversible redox system involving two electrons. As in the case of the vanillic acid, this peak separation was larger in the case of CNT-CPE and smaller for μS-CPE suggesting again that the μS -CPE electrodes are closer to the ideal behavior.

Two irreversible anodic processes were observed when electrodes were immersed in gallic acid (a triphenol) indicating that the oxidation product participates in further chemical reactions or is not reduced at the carbon paste electrode surface (Figures 4 g,h,i). This result could be related with the high reactivity of the produced o-quinones that may condense with other gallic acid molecules through a Michael type addition, yielding purpurogallin-β-carboxylic acid [31]. μS-CPE and G-CPE electrodes showed |E_pa_ − E_pa/2_| values close to the theoretical values for two electron process; again, the CNT-CPE electrochemical behavior was non-ideal.

For ascorbic acid and glutathione, the electrochemical parameters indicated that irreversible processes take place at the electrode surface. The CVs registered using CPEs immersed in ascorbic acid solution were characterized by a broad oxidation peak at ca. 0.38, 0.44 and 0.63 V for μS-CPE, G-CPE and CNT-CPE respectively (Figures 4 j,k,l). According to the literature, this peak can be associated to the oxidation of ascorbic acid involving two electrons and two protons to produce dehydroascorbic acid [12]. According to the potential data, the oxidation is facilitated at the μS-CPE surface that shows a certain degree of electrocatalytic effect. Also in good agreement with the literature, the oxidation of glutathione at the CPEs occurred irreversibly with a peak potential at ca. 1.05 V (Figures 4 m,n,o) [32]. At that potential, the reduced glutathione (GSH) is electrochemically converted to oxidized glutathione (GSSG). In the reduced state, the thiol group of cysteine is able to donate a reducing equivalent (H^+^ + e^−^). In donating an electron, glutathione itself becomes reactive, but readily reacts with another reactive glutathione to form glutathione disulfide (GSSG). Such a reaction is possible due to the relatively high concentration of glutathione in solution analyzed [32].

In conclusion, the electrochemical responses of electrodes constructed from different carbonaceous materials towards the same antioxidant follow similar trends. However, important differences are observed when using different carbon materials. The peak characteristics depend on nature of carbonaceous material used for construction of the electrodes. For all antioxidants analyzed the order of the peak intensities decrease in order μS-CPE > G-CPE > CNT-CPE. The peaks are sharper in the case of μS-CPE and broader in the case of CNT-CPE. The peak potentials observed appears at lowest value with μS-CPE and the highest with CNT-CPE. μS-CPE characteristics calculated (ΔE, |E_pa_ − E_pa/2_|, and i_c_/i_a_) are nearest to the theoretical values. Then, μS-CPE has better performance characteristics comparative with other carbon-based electrodes.

### 3.4. Kinetics of the Responses

In order to establish whether the mechanism of the electrochemical responses was diffusion controlled, experiments were carried out at different scan rates (from 0.01 to 1.0 Vs^−1^) using the three types of electrodes. The next paragraphs illustrate the results obtained when immersing the electrodes in catechol.

**Figure 5.**
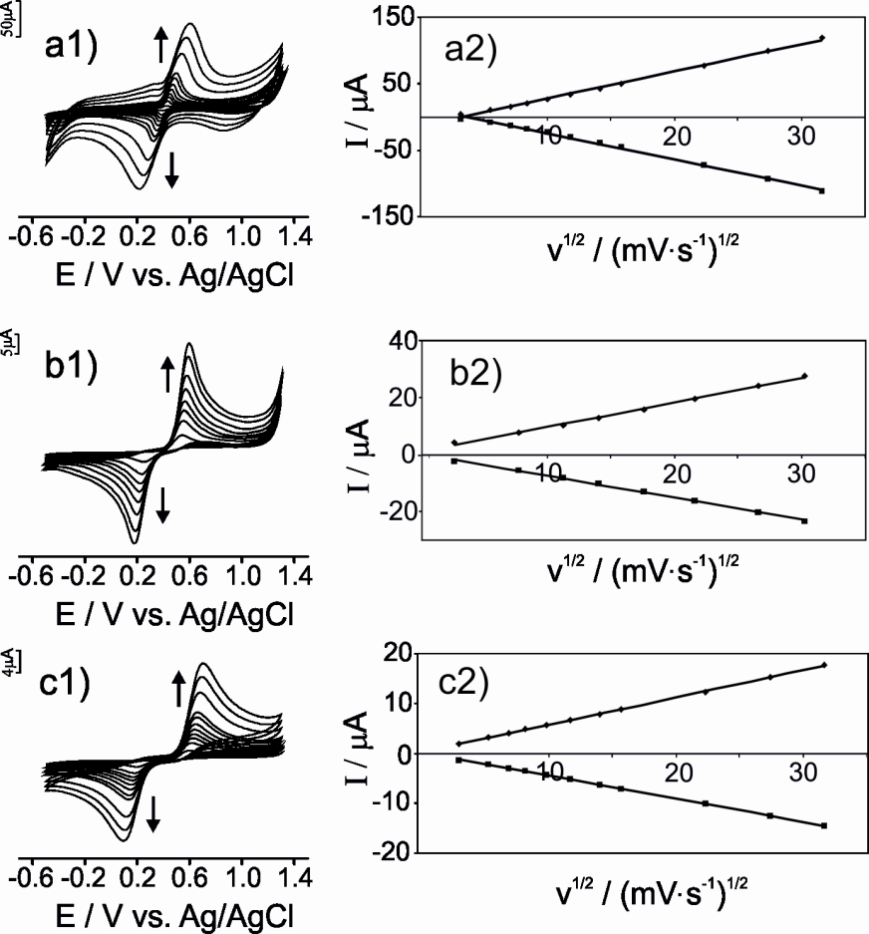
Cyclic voltammograms of: **(a1)** μS-CPE; **(b1)** G-CPE; **(c1)** CNT-CPE in 4 × 10^−4^ mol L^−1^ catechol (pH 3.6) registered with different scan rates (0.01−1.0 Vs^−1^). **(a2); (b2); (c2)** Plots of I_pa_ versus *v*^1/2^, respectively I_pc_ versus v^1/2^.

As observed in Figure 5, the intensity of the peaks and their separation increase with the scan rate (Figure 5 a1, b1, c1). By plotting the peak current versus the square root of the scan rate (*v*^1/2^), a linear relationship is observed in the range from 0.01 to 1.0 Vs^−1^ for both the anodic and the cathodic peak (Figure 5 a2, b2, c2), confirming that the electrochemical process is diffusion controlled. The slopes of the curves follow the trend: μS-CPE > G-CPE > CNT-CPE, indicating that the electron transfer is faster (by one order of magnitude) in the μS-CPE than in CNT-CPE.

The heterogeneous charge-transfer rate constants, k^0^, were calculated from the ΔE values according to the procedure described by Nicholson [33,34], for vanillic acid and catechol. These ΔE values were introduced in a working curve for obtaining the transfer parameter, ψ, and then the k^0^ value for the electron transfer process using the previous equation described by Ramamurthy [35]. According to this equation, linear relationships between ψ and v^−1/2^ were obtained. From the slope of the curves, k^0^ values were calculated.

The heterogeneous electron transfer rate constants determined on the CPEs increased in order CNT-CPE < G-CPE < μS-CPE. These results confirm that in the case of carbon microspheres the structure of carbonaceous material favors the electron transfer.

Kinetic studies can also offer information about the diffusion coefficient of the electroactive compounds. The diffusion coefficients were calculated according to Equation (1). Again the highest D was observed in electrodes prepared from μS-CPE and the lowest for CNT-CPE. The calculations presented in the above paragraphs were also carried out for rest of antioxidants. The results obtained for all antioxidants using CPEs are summarized in Table 2.

The trends observed when immersing the electrodes in catechol were confirmed when analyzing other antioxidants. μS-CPE presents a behavior almost ideal demonstrating a fast electron transfer between the antioxidants and the carbon microspheres and the fastest diffusion coefficients. When the carbon nanotubes were used as electrode material, the electron transfer was difficult and the signals showed a smaller intensity. Moreover, the electrochemical behavior was irreversible. The G-CPE showed an intermediate behavior.

### 3.5. Calibration Curves and Detection Limits

**Figure 6.**
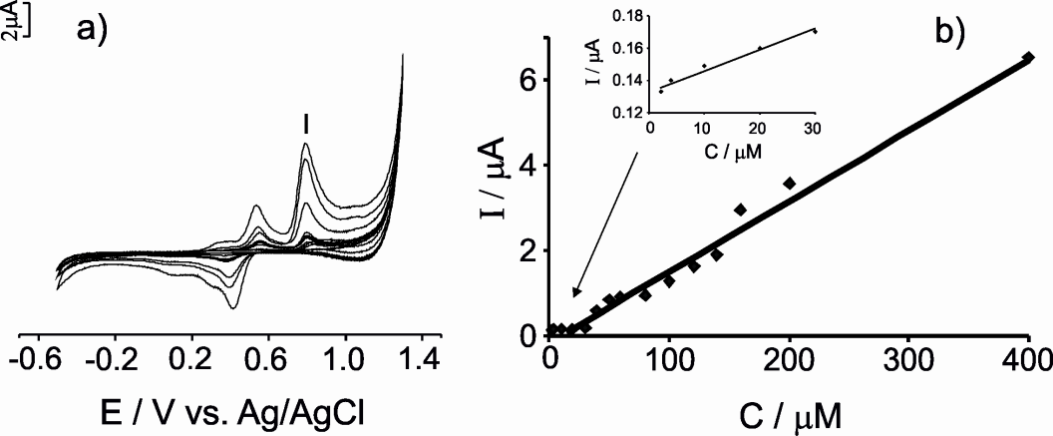
**(a)** Cyclic voltammograms of G-CPE immersed in vanillic acid solutions (10 to 400 μM.); **(b)** Plot of the intensity of anodic peak (I) *vs.* the vanillic acid concentration. Inset: The inset shows the plot of the intensity of anodic peak (I) *vs.* the vanillic acid concentration in 0–30 μM range.

The detection limits attained using the three types of electrodes were calculated from the cyclic voltammograms registered when immersing the CPE electrodes in 10–400 μM solutions of antioxidants prepared in artificial wine solution (pH = 3.6) as supporting electrolyte. The scan rate was 0.1 V·s^−1^ and the potential ranged between −0.5 V and 1.3 V. The detection limits (LOD) were calculated by representing the intensity of the most intense anodic peak (see Figure 2, peak I) *vs.* the concentration of the corresponding antioxidant according to the 3s_b_/m criterion, where m is the slope of the calibration graph, and s_b_ was estimated as the standard deviation (n = 7) of the intensity of the peak from different solutions of the corresponding antioxidant at the concentration level corresponding to the lowest concentration of the calibration plot. The responses at different concentrations and the calculations of the LOD are illustrated in Figure 6 for vanillic acid measured with a G-CPE (concentrations ranging from 10 to 400 μM).

As observed in (Figure 6a), the intensity of the anodic peaks increased with vanillic acid concentration. A linear response was observed in the 10–400 μM range (Figure 6b), with sensitivity of 0.0166 μA·μM^−1^. The LOD calculated was 2.85 μM. The detection limits and the sensitivity calculated for all the antioxidants measured with the all three electrodes are summarized in Table 3.

The detection limits are in the range of the detection limits published for carbon based electrodes [36] and are in the range of the polyphenol index commonly found in foods [37]. The LODs calculated were similar for all CPEs; in general, G-CPE present smallest LODs for all antioxidants studied. These results could be related with the interaction between the antioxidants and electrodes and the background current. For this reason, the high conductivity of the μS-CPE makes difficult the detection of antioxidants al low concentration levels.

### 3.6. Reproducibility

The reproducibility of the results was examined by successive seven measurements of 4 × 10^−4^ mol·L^−1^ catechol using the optimum conditions mentioned above. The relative standard deviation (RSD) of anodic peak potential was calculated and it was found to be 2.9% for G-CPE, 3.2% for μS-CPE, and 3.9% for CNT-CPE.

The reproducibility of the electrode construction was also examined by the determination of 4 × 10^−4^ mol·L^−1^ catechol using three electrodes prepared from the same carbon paste. RSD of anodic peak potential was found to be less than 4% for all carbon types used, which indicated that this method give a good reproducibility.

### 3.7. Carbon Electrodes Array Data Treatment

With the objective to evaluate the capability of the carbon paste electrodes to discriminate among antioxidants, an array formed by the three electrodes was constructed and Principal Component Analysis was conducted using the electrochemical signals as the input variable. Six replicates of each measurement were carried out.

**Figure 7.**
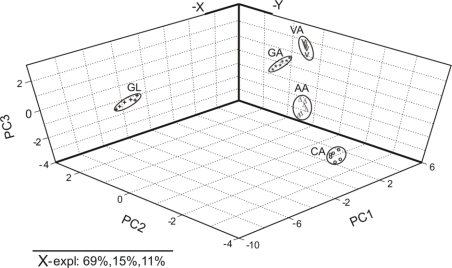
3D score plot of PCAs of CPEs exposed to antioxidants. (GL—glutathione; AA—ascorbic acid; VA—vanillic acid; CA—catechol; GA—gallic acid).

Figure 7 shows the PCA scores plot of the principal components (PC1 versus PC2 versus PC3). The PC1 accounts for the 69% of the variation in the electrochemical signal, the PC2 account for the 15% and the PC3 for the 11%. Overall, first, second and third principal components explained 95% percent of the total variance between the samples.

As observed in Figure 7, the antioxidants appear grouped into two clusters: the phenols and ascorbic acid are situated on the right side of the diagram, whereas glutathione is located on the left part of the graph. Ascorbic acid is included in the clusters corresponding phenolic compounds; this result is in good agreement with chemical structure of ascorbic acid that have two hydroxyl groups with a behavior similar of those presents in phenolic compounds. The oxidation of ascorbic acid, as well as, of phenolic compounds gave quinonic compounds in a two electron process. The PCA scores plot structure is in agreement with the electrochemical signals registered for each antioxidant. The results indicate that it is clearly possible to discriminate antioxidants in function of chemical structure.

## 4. Conclusions

Carbon paste electrodes made from different carbonaceous materials were characterized as voltammetric sensors for detection of antioxidants such as ascorbic acid, glutathione and phenolic compounds (mono-, di- and triphenols). The CPEs showed characteristic peaks associated with the redox electrochemical processes of the antioxidants, strongly related with chemical structures. On the other hand, the shape and position of the peaks are influenced by nature of carbonaceous materials used for electrode construction. The intensity of the signals decreases in the following order: μS-CPE > G-CPE > CNT-CPE; the reversibility criteria are nearest to the theoretical values for μS-CPE. The kinetic studies demonstrate that electrochemical behavior of antioxidants at CPEs surfaces is diffusion controlled. The heterogeneous electron transfer rate constants determined by the Nicholson method increase in the order: CNT-CPE<G-CPE<μS-CPE. The detection limits were in the range of 2.85 μM–16.35 μM for antioxidant solutions. Application of the principal component method for the analysis of the cyclic voltammograms obtained for CPEs immersed in antioxidant solutions shows a 100% discrimination of the samples in function of chemical structure.

## Figures and Tables

**Table 1.** Electrochemical parameters obtained from the responses of the CPEs towards antioxidants at 0.1 V·s^−1^ in 12% ethanol, 0.05 mol·L^−1^ tartaric acid, and NaOH (pH 3.6).

**G-CPE**									
**Antioxidant**	E_pa_/V	E_pc_/V	ΔE/V	E_1/2_/V	(E_pa_−E_pa/2_) / V	(E_pa_ + E_pa/2_)/2 / V	i_pa_/μA	i_pc_/μA	i_pc_/i_pa_
Vanillic acid	0.53	0.43	0.10	0.48	0.05	0.50	3.00	−3.68	1.22
	0.79	-	-	-	0.05	0.76	8.13	-	-
Catechol	0.59	0.21	0.38	0.39	0.07	0.55	6.16	−4.48	0.73
Gallic acid	0.45	-	-	-	0.04	0.44	4.2	-	-
	0.81	-	-	-	-	-	3.73	-	-
Ascorbic acid	0.45	-	-	-	0.08	0.41	1.78	-	-
Glutathione	1.12	-	-	-	0.08	1.08	4.48	-	-
**μS-CPE**									
**Antioxidant**	E_pa_/V	E_pc_/V	ΔE/V	E_1/2_/V	(E_pa_−E_pa/2_) / V	(E_pa_ + E_pa/2_)/2 / V	i_pa_/μA	i_pc_/μA	i_pc_/i_pa_
Vanillic acid	0.49	0.46	0.04	0.48	0.04	0.47	3.23	−2.73	0.84
	0.78	-	-	-	0.05	0.75	6.55	-	-
Catechol	0.45	0.35	0.10	0.41	0.06	0.43	26.4	−23.43	0.88
Gallic acid	0.43	-	-	-	0.04	0.41	6.04	-	-
	0.77	-	-	-	-	-	3.84	-	-
Ascorbic acid	0.39	-	-	-	0.19	0.29	2.73	-	-
Glutathione	1.11	-	-	-	0.14	1.04	5.12	-	-
**CNT-CPE**									
**Antioxidant**	E_pa_/V	E_pc_/V	ΔE/V	E_1/2_/V	(E_pa_−E_pa/2_) / V	(E_pa_ + E_pa/2_)/2 / V	i_pa_/μA	i_pc_/μA	i_pc_/i_pa_
Vanillic acid	0.65	0.06	0.59	0.35	0.09	0.60	0.63	−0.72	1.14
	0.90	0.78	0.12	0.84	0.10	0.85	2.1	0.08	-
Catechol	0.64	0.15	0.48	0.40	0.09	0.59	5.78	−4.37	0.76
Gallic acid	0.57	-	-	-	0.11	0.51	0.97	-	-
	0.86	-	-	-	-	-	1.07	-	-
Ascorbic acid	0.64	-	-	-	0.19	0.54	1.18	-	-
Glutathione	1.08	-	-	-	0.10	1.03	2.64	-	-

**Table 2.** The results obtained in the kinetic studies for all CPEs in antioxidant solution.

**Electrode**	**Antioxidant**	**Regression equation I_pa_*vs.* v^1/2^ (Anodic)**	**Regression equation I_pc_*vs.* v^1/2^ (Cathodic)**	**k^0^ (cm·s^−1^)**	**D /cm^2^·s^−1^**
**μS-CPE**	Vanillic acid	y = 0.6171x − 2.5289 R^2^ = 0.9791	y = −0.1832x + 0.4008 R^2^ = 0.9672	0.066	1.11 × 10^−6^
	Catechol	y = 4.0748x − 12.989 R^2^ = 0.9973	y = −3.8849x + 13.959 R^2^ = 0.9961	0.0236	6.45 × 10^−6^
	Gallic acid	y = 0.5832x − 1.2836 R^2^ = 0.9419	-	-	1.32 × 10^−7^
	Ascorbic acid	y = 0.8451x − 4.8077 R^2^ = 0.9524	-	-	2.77 × 10^−7^
	Glutathione	y = 0.7143x − 4.717 R^2^ = 0.9712	-	-	1.58 × 10^−6^
**G-CPE**	Vanillic acid	y = 1.3054x − 5.4805 R^2^ = 0.9687	y = −0.8723x + 4.1084 R^2^ = 0.9803	0.099	2.45 × 10^−6^
	Catechol	y = 1.8699x − 2.4293 R^2^ = 0.9968	y = −1.6633x + 3.4139 R^2^ = 0.9977	0.0259	5.04 × 10^−6^
	Gallic acid	y = 0.126x + 1.9363 R^2^ = 0.9523	-	-	2.29 × 10^−8^
	Ascorbic acid	y = 0.0765x + 0.7815 R^2^ = 0.9899	-	-	8.44 × 10^−7^
	Glutathione	y = 0.3594x + 0.3241 R^2^ = 0.9835	-	-	1.49 × 10^−6^
**CNT-CPE**	Vanillic acid	y = 0.6171x − 2.5289 R^2^ = 0.9791	y = −0.1832x + 0.4008 R^2^ = 0.9672	0.0039	6.74 × 10^−7^
	Catechol	y = 0.5522x + 0.0976 R^2^ = 0.9998	y = −0.4658x + 0.2345 R^2^ = 0.9996	0.0031	5.40 × 10^−7^
	Gallic acid	y = 0.1465x + 0.8831 R^2^ = 0.9257	-	-	8.33 × 10^−9^
	Ascorbic acid	y = 0.0531x + 0.318 R^2^ = 0.9414	-	-	4.99 × 10^−9^
	Glutathione	y = 0.1873x + 2.4388 R^2^ = 0.9943	-	-	4.96 × 10^−7^

**Table 3.** Sensitivities and LODs resulted from calibration curves i_pa_ *vs*. c.

**Electrode**	**Antioxidant**	**Sensitivity/μA·μM^−1^ (Anodic peak I)**	**R^2^**	**LOD / μM**
**G-CPE**	Vanillic acid	0.0166	0.9613	2.85
	Catechol	0.0155	0.9937	6.32
	Gallic acid	0.0062	0.9893	11.32
	Ascorbic acid	0.0051	0.9798	9.30
	Glutathione	0.0106	0.985	4.47
**μS-CPE**	Vanillic acid	0.0175	0.9712	3.82
	Catechol	0.1099	0.9659	9.83
	Gallic acid	0.0018	0.9141	13.24
	Ascorbic acid	0.0018	0.9141	16.35
	Glutathione	0.0238	0.9619	6.32
**CNT-CPE**	Vanillic acid	0.0005	0.9534	4.13
	Catechol	0.0003	0.9497	5.47
	Gallic acid	0.0097	0.96	13.26
	Ascorbic acid	0.0059	0.9159	10.34
	Glutathione	0.0075	0.9581	5.48
